# Microbial Musings – February 2020

**DOI:** 10.1099/mic.0.000901

**Published:** 2020-02-28

**Authors:** Gavin H. Thomas

**Affiliations:** ^1^​ Department of Biology, University of York, YO10 5YW, UK

Microbiology has been at the forefront of many people’s minds during February as the new coronavirus outbreak is tracked on a global scale with major efforts to control its spread. Daily updates on infection numbers, transmission rates, genome sequences and efforts to develop rapid diagnostics and a vaccine are keeping our field in the news. However, despite the unprecedented gathering and sharing of knowledge, stopping the spread of the virus still seems almost impossible in our global 21st-century world. You can keep up to date with news from the Microbiology Society (@MicrobioSoc) or follow legendary US microbiologist Richard Lenski’s (@RELenski) blog (https://telliamedrevisited.wordpress.com/).

For this month’s musings we start with a couple of articles about bacterial metabolism. The first is an interesting and novel review of metabolic networks found in the human gut microbiome [1]. It is novel in that it was written as a collaborative effort by a consortium of undergraduate students studying microbiology at Concordia University in Montréal, Canada, led by Chiara Gamberi (@ChiaraGamberi), following a model that she used in 2019 [2]. Using such a large cohort, with students split into small teams working on particular subtopics, the review is both extensive and very broad ranging in its coverage of the metabolic interactions of the gut microbes with the host and their functions in health and disease. Professor Gamberi spoke to the Society about this paper and its pedagogical underpinnings, which you can read about on the Microbiology Society website.

The second metabolic network-related paper is a primary article from the lab of Bernard Palsson at UCSD (@ucsd_sbrg). His group has led the field in creating and using whole-genome metabolic analysis for fundamental biological study and biotechnological applications [3]. In our lab, where *Escherichia coli* K-12 is a workhorse organism of study, the names of members of his group are legendary, as the models are named after their creators (author's initials and the number of genes in the mode). Hence, the models of Jennifer Reed (iJR904) (@UWMadCBE), Adam Feist (iAF1260) (@DTUBiosustain) and later Jeffrey Orths (iJO1366) [4–6] have been used as starting points for many of our own studies [7, 8]. More recently Feist and Palsson have been using experimental evolution or adaptive laboratory evolution (ALE) as an approach to push the boundaries of *E. coli* metabolism and generally showing its remarkable robustness and flexibility [9]. In this work they use ALE to understand more about the response of *E. coli* K-12 MG1655 to acid stress induced by growth at pH 5.5 in defined glucose minimal medium [10]. The ability of *E. coli* strains to be able to resist quite strong acid for short periods of time likely reflects the necessity for this capability to enable passage through the stomach (pH 2) and later survival in the more weakly acidic intestine of its host, and here a weaker acid stress (pH 5.5) was applied for 800 generations. Of the six evolved lines, five had mutations in *rpoC*, encoding the beta-subunit of RNA polymerase, which they conclude, after measuring changes in gene expression in the evolved lines by RNAseq, are due to generally increased fitness leading to faster growth, rather than any particular improvement in acid resistance. A related study from the lab of acid-stress aficionado Joan Slonczewski found a similar mutation in RNA polymerase subunits in a study at external pH 4.8 [11], but also additional mutations removing acid resistance systems such as the lysine decarboxylase (CadA) needed for extreme acid resistance, suggesting that adaptation to milder acid stress over long periods leads to a tempering of the extreme acute response. This reinforces the findings from ALE that you really get what you select for, obvious as this sounds, and so small differences in experimental design can lead to very different outputs. Interested readers in the European Union (EU) can engage with the new COST Action on EuroMicropH – ‘Understanding and exploiting the impacts of low pH on micro-organisms’ (@EuroMicropH) led by action Chair Pete Lund (@lundpa) from Birmingham, UK.

Next we have a couple of papers continuing last month’s theme on the genus *Pseudomonas *. This month we start with a species not covered in January, *Pseudomonas putida *, an organism with great potential for use in biotechnology due to its robust constitution and interesting biochemistry [12]. Within the EU this bug is being developed through large projects such as EmPowerPutida (@EmPowerPutida), with strong advocates like Victor de Lorenzo in Madrid, Spain (@vdlorenzo_CNB). The work is this issue is from Cecilia Arraiano’s group (@ArraianoLab) in Lisbon, Portugal (@itqbunl) and Ralf Takors in Stuttgart, Germany, who investigated patterns of gene expression when growing *P. putida* KT2440 in a chemostat with a modification called a plug flow reactor [13]. Here, after reaching steady state in the chemostat, portions of the culture are flowed around a loop system where the lack of new media addition causes rapid depletion of glucose, leading to transient changes in gene expression before the cells flow back into the chemostat. In this plug flow design samples are taken at different distances around the loop and RNA profiles are measured by RNAseq, with the glucose-replete chemostat functioning as the comparison. Using their experimental data as the starting point, they combined this with *in silico* prediction tools for small non-coding RNAs (ncRNAs) and discovered a set of 725 ncRNAs. This new set of detectable ncRNAs is not only very large, but remarkably only a small number match known ncRNAs, and a related study from Katherine Long’s group at the Technical University of Denmark (DTU) near Copenhagen, Denmark, examining different stresses found a quite different set of transcripts [14], suggesting that there is much to learn about the diversity and functioning of these short transcripts in cell physiology.

Our second *Pseudomonas* paper continues a theme from last month on the role of soluble secreted metabolites acting as signalling molecules [15]. In the new study, from Jerry Reen and Fergal O’Gara’s labs in Cork, Ireland, they examine how secreted alkyl-quinolones of related structures have significantly different effects on other bacterial pathogens [16]. Using members of the *Vibrionaceae *, they show activity against *Vibrio cholerae* and *Vibrio vulnificus *, both human pathogens, but that *Vibrio parahaemolyticus* is totally resistant. The authors remind us that the *Pseudomonas *–* Vibrio* interaction has attracted a lot of attention through the discovery of ‘tit-for-tat’ killing via type VI secretions systems (T6s) [17], but here secreted small molecules are also clearly important for this inter-species competition.

The group of Jake McKinlay (@JakeMcKinlay and @mckinlab) at Indiana University, Bloomington, USA have published an interesting paper, also highlighted by Senior Editor Professor Gail Preston as Editor’s Choice for the February issue, demonstrating robustness in how bacterial cells manage their redox state [18]. The photosynthetic bacterium *Rhodospirillum rubrum *, when growing in the light with an organic source of carbon, which we know as photoheterotrophic growth, generates an excess of reducing potential that it must dissipate. Classically, this bug uses the Calvin cycle to reoxidize its reduced electron carriers, but in this paper the group remove this system to see how the bugs cope. By using labelled carbon (^13^C), they show that the cells run part of their tricarboxylic acid (TCA) cycle backwards to provide precursors for alternative pathways to synthesize some amino acids. When the authors added these amino acids to the media (so the bugs did not need to make them), the Calvin cycle mutant couldn’t grow. Hence, they concluded that the physiology of this microbe had built-in metabolic robustness to deal with varying redox stresses through multiple routes.

For the last paper in this month’s musings we move to a very cool eukaryotic microbe, the soil amoeba *Dictyostelium discoideum*. As a bacteriologist it is easy to forget how incredibly interesting the cell biology of these microbes is. While some bacteria, such as *Myxococcus *, can swarm together, differentiate and form fruiting bodies, this pales into insignificance compared to the accomplishments of ‘Dicty’, as it is known to its researchers. Initiation of this process involves the secretion of cyclic AMP (cAMP), which is used as a signal for chemotaxis of cells to form the initial multicellular aggregate. An important regulator of cAMP levels is a phosphodiesterase called RegA and in this study from the group of Jeff Hadwiger at Oklahoma State University, USA, including Nick Kuburich (@Kubu59), the authors examine how the MAP kinase Erk2 interacts with RegA, as it is known that the phosphorylation of a site on RegA regulates its function negatively [19]. Using genetic and biochemical experiments, they find evidence for a clear interaction between the proteins. The study supports the idea that MAP kinase regulation of cAMP signalling is conserved across diverse eukaryotes. The Microbiology Society will be running a symposium on protists, co-sponsored by Protistology UK, called ‘Exploring the eukaryotic tree of life’ at Annual Conference this year, so come and learn more about these fascinating microbes. Early bird discount ends on 3 March, so sign up now if have not not already for this great event.

Gavin Thomas, Deputy Editor-in-Chief.
